# Hématome hépatique néonatal: à propos d’un cas

**DOI:** 10.11604/pamj.2017.27.15.11900

**Published:** 2017-05-08

**Authors:** Hanae Harchaoui, Bousayna Iraqi, Houria knouni, Youness Taboz, Hasnae Benkirane, Hassan Aguenaou, Amina Barkat

**Affiliations:** 1 Equipe de recherche en santé et nutrition du couple mère enfant, faculté de médecine et de pharmacie de Rabat, université Mohamed V- RABAT, Service de médecine et réanimation néonatales, Centre Hospitalier Ibn Sina, Rabat, Maroc; 2 Université Ibn Tofail, Unité Mixte

**Keywords:** Hématome, foie, nouveau-né, traumatisme obstétrical, Hematoma, liver, new-born, obstetrical trauma

## Abstract

L'hématome hépatique est une affection méconnue, peut être gravissime et son diagnostic se fait souvent lors de l'autopsie périnatale. Devant une symptomatologie clinique, souvent, non spécifique et un tableau biologique retardé, le diagnostic est basé essentiellement sur l'échographie et le traitement est souvent conservateur. Nous rapportons un cas d'hématome hépatique chez un nouveau-né à terme, asymptomatique dans un contexte de traumatisme obstétrical.

## Abstract (en)

Hepatic hematoma is a condition of unrecognized origin which can become extremely serious and whose diagnosis is often made during perinatal autopsy. It is often characterised by nonspecific clinical manifestations and a long delay before biological presentation.The diagnosis is essentially based on ultrasound and treatment is often conservative.We here report a case of a full-term newborn with asymptomatic hepatic haematoma due to obstetrical trauma.

## Introduction

L'hématome hépatique est une affection méconnue, peut être gravissime et son diagnostic se fait souvent lors de l'autopsie périnatale [1]. Son incidence varie entre 2,8 et 15% selon les différentes séries de décès néonataux [2–4]. Il est habituellement sous-capsulaire. Dans certains cas, l'hématome hépatique peut être associé à une rupture du foie et/ou un hémopéritoine [5]. Plusieurs étiologies ont été décrites à savoir: les traumatismes obstétricaux, les coagulopathies et la septicémie [5].

## Patient et observation

Nous rapportons un cas d'hématome hépatique chez un nouveau-né à terme, sans signes cliniques spécifique et dans un contexte de traumatisme obstétrical néonatale.

**Figure 1:**
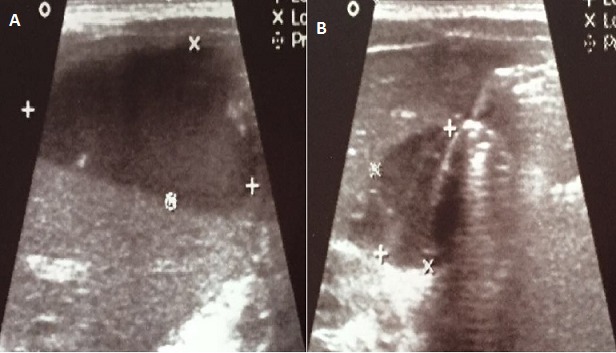
Aspect échographique en faveur d’un hématome sous capsulaire gauche 35×17cm et un hématome latéro-vésiculaire droit mesurant 17×13cm

**Figure 2:**
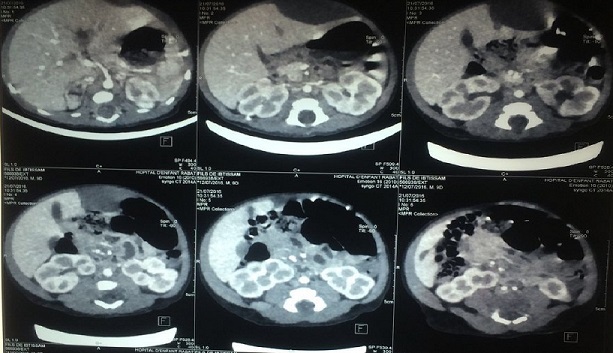
Aspect scannographique montrant une thrombose portale partielle avec hématome sous capsulaire gauche et de la surrénale droite

**Figure 3:**
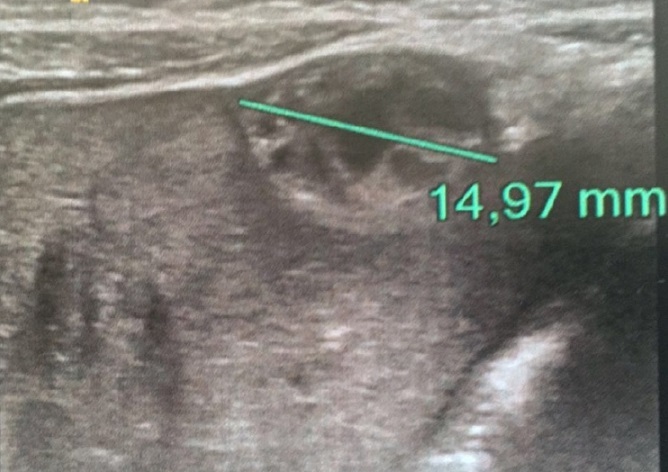
Echographie de contrôle à 1 mois de vie montrant un hématome sous capsulaire en avant du segment V et II hépatiques mesurant 15mm de grand axe

**Observation**: Il s'agissait d'un nouveau-né de sexe masculin. Issu d'une mère de 25 ans, multipare, sans antécédents pathologiques particuliers. L'interrogatoire ne révélait pas de notion d'hémophilie dans la famille. La grossesse avait été suivie, de déroulement normal et menée à terme. Pas de prise médicamenteuse durant toute la grossesse. L'accouchement par voie basse, dystocique, ayant nécessité une extraction par ventouse, d'un nouveau né macrosome (poids de naissance de 4250g), dans un état de mort apparente (APGAR 3-3-4/10). L'enfant a été réanimé pendant 20 minutes à la salle d'accouchement (intubation endo-trachéal et ventilation au masque) avec bonne évolution. Administration de vitamine K à la naissance. L'examen neurologique à l'admission montrait un nouveau-né hypotonique, des pupilles égales et réactives avec une énorme bosse séro-sanguine. L'examen cardiovasculaire et pleuro-pulmonaire étaient sans particularités, ainsi que l'examen abdominal. Après stabilisation du patient: monitorage, mise en place d'une voie veineuse, réalisation d'un bilan sanguin, administration du phénobarbital en dose de charge puis dose d'entretien, bolus de calcium pendant trois jours, antalgique et antibiothérapie, un bilan radiologique a été réalisé. La radiographie du thorax et l'échographie trans-fontanellaire étaient normales. L'électroencéphalogramme précoce en faveur d'une encéphalopathie anoxo-ischémique sévère. La TDM cérébrale a montrée des lésions anoxo-ischémique de la substance blanche et des noyaux gris centraux. L'échographie abdominale objectivait un foie augmenté de taille, un hématome sous capsulaire gauche mesurant 35×17cm et un hématome latéro-vésiculaire droit mesurant 17×13cm (Figure 1). La TDM abdominale, réalisée à 15 jours de vie, montrait une thrombose portale partielle avec hématome sous capulaire gauche et de la surrénale droite (Figure 2). Les résultats du bilan biologique montraient : un taux d'hémoglobine à 12,5 g/dL; un taux des plaquettes à 323000/mm^3^; un bilan rénal et hépatique normal; un TP à 100 %; un TCK normal; le taux des facteurs V, VII, VIII et IX ainsi que le fibrinogène étaient normaux ; et une protéine C réactive élevée (CRP) à 47 mg/L. L'évolution clinique était favorable et le nouveau-né est sorti sous traitement anticonvulsivant. À 1 mois de vie, l'échographie de contrôle a montré la diminution de la taille de l'hématome hépatique sous capsulaire avec régression de l'hématome latéro-vésiculaire (Figure 3).

## Discussion

Le processus de l'accouchement est un ensemble de forces de compressions, de contractions, et de tractions. Lorsque la taille du foetus, la présentation ou l'immaturité neurologique compliquent cet événement, ces forces peuvent conduire à des lésions obstétricales néonatales. Malgré la nette diminution de leur incidences en raison de l'amélioration de la prise en charge obstétricale et du diagnostic prénatal, les traumatismes obstétricaux restent une cause importante de la morbi-mortalité néonatale [6]. L'hématome sous capsulaire du foie correspond à une collection hématique sous la capsule de Glisson, qui doit être distinguée de l'hématome intra-parenchymateux, où le saignement est moins important. Bien que ce soit une affection fréquente chez l'adulte, l'hématome sous capsulaire reste une complication inhabituelle des traumatismes à la naissance [6]. Trois mécanismes sont possibles : le traumatisme direct, la compression du thorax contre la surface du foie et la rupture des insertions ligamentaire du foie [2]. Les étiologies de l'hématome hépatique chez le nouveau né sont multiples et peuvent être classées en causes obstétricales qui regroupent l'accouchement dystocique, la présentation du siège et le travail trop rapide ; causes néonatales dominées par la prématurité, le faible poids de naissance, l'hypoxie, les coagulopathies, la septicémie, la pose d'un cathéter ombilical, la ventilation mécanique, la réanimation néonatale et l'hépatomégalie (en rapport avec une érythroblastose, infection congénitale, nouveau-né de mère diabétique…), et les causes maternelles à savoir l'âge maternel avancé, la multiparité, l'éclampsie, l'hématome rétro-placentaire… [1, 3]. La notion de traumatisme à la naissance et l'utilisation de la ventouse restent des causes majeures de survenue d’hématome hépatique, même en l'absence d'autres facteurs de risque, et c'est le cas dans notre contexte, Cela peut être expliqué par le fait que la pression négative importante exercée par la ventouse peut conduire à des changements significatifs de pressions entres les différentes cavités du corps, y compris le système veineux. Ces pressions peuvent être transmises au foie et causer une hémorragie et/ou un hématome hépatique[6]. La tableau clinique dépend du degré de la perte sanguine. Les hématomes sous capsulaires peuvent se présenter dans un tableau insidieux avec une anémie progressive, un ictère, une irritabilité ou une détresse respiratoire. Il s'agit rarement d'une masse abdominale isolée et peut mimer une tumeur du foie. Cependant, ils peuvent augmenter progressivement leurs volumes afin de se rompre et être à l'origine d'une détérioration aigue. L'hématome scrotal a été décrit comme signe révélateur [1, 6]. Dans notre cas, le patient était asymptomatique. L'échographie abdominale est l'examen de choix. Elle pose facilement le diagnostic d'hématome sous capsulaire. Elle permet d'exclure sa rupture dans la cavité péritonéale, de faire le diagnostic différentiel avec une pathologie néoplasique du foie et d'assurer le suivi à long terme [6]. L'utilité de la TDM abdominale est discutable, mais elle permet de mieux étudier les lésions hépatiques détectées à l'échographie et de préciser leurs étendus et leurs âge [3]. Le traitement commence par la correction de l'état hémodynamique et d'un éventuel trouble de coagulation associé. Si le nouveau-né est stable le traitement est conservateur. En cas de rupture ou d' instabilité hémodynamique, laparotomie est nécessaire pour contrôler le saignement [3, 7]. Les traumatismes du foie doivent être suspectés chez tout nouveau-né avec un facteur de risque prédisposant qu'il soit maternel, fœtal ou lié à l'accouchement ; une anémie aigue ou un état de choc d'étiologie incertaine. Il s'agit d'une maladie potentiellement fatale et la détection précoce reste le meilleur moyen pour réduire les complications.

## Conclusion

L'hématome sous capsulaire du foie est une complication rare et grave qui peut être mortelle. Il peut survenir dans un contexte de traumatismes obstétricaux et présenter des symptômes atypiques. L'échographie est d'un grand apport et la prise en charge est conservatrice.

## Conflits d’intérêts

Les auteurs ne déclarent aucun conflit d'intérêts.
